# Repeated dexamphetamine treatment alters the dopaminergic system and increases the phMRI response to methylphenidate

**DOI:** 10.1371/journal.pone.0172776

**Published:** 2017-02-27

**Authors:** Anouk Schrantee, Jordi L. Tremoleda, Marzena Wylezinska-Arridge, Valentine Bouet, Peter Hesseling, Gideon F. Meerhoff, Kora M. de Bruin, Jan Koeleman, Thomas Freret, Michel Boulouard, Emilie Desfosses, Laurent Galineau, Alessandro Gozzi, François Dauphin, Willy Gsell, Jan Booij, Paul J. Lucassen, Liesbeth Reneman

**Affiliations:** 1 Department of Radiology, Academic Medical Center, University of Amsterdam, Amsterdam, The Netherlands; 2 Biological Imaging Centre, Imperial College London, White City, London, United Kingdom; 3 Centre for Trauma Sciences, The Blizard Institute, London, United Kingdom; 4 Neuroradiological Academic Unit, Department of Brain Repair and Rehabilitation, UCL Institute of Neurology, Queen Square, London, United Kingdom; 5 Normandie-Université, GMPc, EA 4259, Université de Caen Basse-Normandie, Caen, France; 6 Swammerdam Institute for Life Sciences, Center for Neuroscience, University of Amsterdam, Amsterdam, The Netherlands; 7 Department of Nuclear Medicine, Academic Medical Center, University of Amsterdam, Amsterdam, The Netherlands; 8 UMR Inserm U930, Université François-Rabelais de Tours, Tours, France; 9 Functional Neuroimaging Laboratory, Istituto Italiano di Tecnologia, Center for Neuroscience and Cognitive Systems @ UNITN, Rovereto, Italy; 10 Biomedical MRI, Department of Imaging and Pathology, KU Leuven, Leuven, Belgium; Radboud University Medical Centre, NETHERLANDS

## Abstract

Dexamphetamine (AMPH) is a psychostimulant drug that is used both recreationally and as medication for attention deficit hyperactivity disorder. Preclinical studies have demonstrated that repeated exposure to AMPH can induce damage to nerve terminals of dopamine (DA) neurons. We here assessed the underlying neurobiological changes in the DA system following repeated AMPH exposure and pre-treated rats with AMPH or saline (4 times 5 mg/kg s.c., 2 hours apart), followed by a 1-week washout period. We then used pharmacological MRI (phMRI) with a methylphenidate (MPH) challenge, as a sensitive and non-invasive in-vivo measure of DAergic function. We subsequently validated the DA-ergic changes post-mortem, using a.o. high-performance liquid chromatography (HPLC) and autoradiography. In the AMPH pre-treated group, we observed a significantly larger BOLD response to the MPH challenge, particularly in DA-ergic brain areas and their downstream projections. Subsequent autoradiography studies showed that AMPH pre-treatment significantly reduced DA transporter (DAT) density in the caudate-putamen (CPu) and nucleus accumbens, whereas HPLC analysis revealed increases in the DA metabolite homovanillic acid in the CPu. Our results suggest that AMPH pre-treatment alters DAergic responsivity, a change that can be detected with phMRI in rats. These phMRI changes likely reflect increased DA release together with reduced DAT binding. The ability to assess subtle synaptic changes using phMRI is promising for both preclinical studies of drug discovery, and for clinical studies where phMRI can be a useful tool to non-invasively investigate DA abnormalities, e.g. in neuropsychiatric disorders.

## Introduction

Dexamphetamine (AMPH) is a psychostimulant that is often used, both recreationally and for the treatment of attention deficit hyperactivity disorder (ADHD). Shortly after acute administration, dexamphetamine (AMPH) induces large increases in dopamine (DA) concentrations. However, repeated AMPH treatment may cause lasting reductions in striatal DA, in its major metabolite dihydroxyphenylacetic acid (DOPAC), its rate-limiting enzyme tyrosine hydroxylase, its membrane transporter (DAT) and in the vesicular monoamine transporter (VMAT2) [1–4], not only at high (toxic) doses, but likely also at already much lower doses such as those used to treat ADHD patients [5]. In addition to the reduction in DA-ergic presynaptic markers, repeated intermittent exposure to AMPH can result in an exaggerated DA response [6,7], that is thought to be mediated by increased DA release and/or reductions in DA re-uptake and metabolism [7].

Previous studies have demonstrated the potential of pharmacological MRI (phMRI) as a meaningful tool to visualize DAergic dysfunction [8,9]. PhMRI can measure evoked changes in brain hemodynamics as a result of neurotransmitter-specific drug challenges. Previous studies have demonstrated that phMRI can visualize the effects of DA neurotoxicity, which strongly correlated with measures of the DAT, DA concentrations and behaviour [8,10]. For example, DA neuron loss induced by strong and well-documented DAergic neurotoxins, such as 6-hydroxydopamine (6-OHDA) and 1-methyl-4-fenyl-1,2,3,6-tetrahydropyridine (MPTP) resulted in a blunted phMRI response in animals. More recent preclinical studies have demonstrated that phMRI can also visualize more complex alterations in DA-ergic nerve terminals in animal models of addiction [11] and autism [12]. As phMRI is non-invasive, it could potentially be a powerful tool to investigate effects of AMPH treatment on the DA system of children and adolescents with ADHD. Here, we used phMRI to assess remodelling of the DA synapse in a rodent model of repeated AMPH administration [13], known to induce neurotoxic changes to the DA system, using phMRI. In order to further validate the neurobiological substrates underlying changes in phMRI signal, we assessed DAT as well as DA receptor alterations, DA levels and its metabolites using immunocytochemistry, autoradiography, ex vivo storage phosphor imaging and high-performance liquid chromatography (HPLC) analysis.

## Methods

PhMRI was used to assess DA functionality following a DA-ergic challenge with AMPH. In the same rats, immunocytochemistry (ICC) was used afterwards to measure overall DAT and DA receptor levels. In different groups of rats with the same treatment protocol, *in vitro* autoradiography was used to assess DAT and DA D_1_ (DRD1) availability, *ex vivo* storage phosphor imaging to measure striatal DA D_2/3_ receptor (DRD2/3) availability and high-performance liquid chromatography (HLPC) to assess in vivo levels of DA and its metabolites.

### Animal procedures and treatment

All experiments were approved by the local animal ethical committee and carried out in strict accordance with European guidelines (EU Directive 2010/63/EU) to minimize animal suffering. The studies were conducted in adult male Sprague Dawley rats (Charles River, UK, Janvier Labs, France and Harlan, the Netherlands), weighing between 200–300 g. In all experiments, the rats were divided in two groups that received either treatment with AMPH (5 mg/kg s.c. four times 2 hours apart) or with saline (also four times 2 hours apart s.c.), as this dose has been shown to induce damage in the DA synapse [13]. AMPH (Sigma Aldrich) was dissolved in 0.9% saline and injected s.c. in a final volume of 1 ml/kg body weight.

1. For the phMRI and immunohistochemistry, all experiments were carried out in accordance with the Animals Scientific Procedures Act 1986 under a project approved by the United Kingdom Home Office. N = 21 rats were pre-treated with saline and N = 21 were pre-treated with AMPH. Due to the experimental set-up it was not possible to obtain perfused brains for all animals in the phMRI study. Therefore, N = 15 AMPH pre-treated and N = 15 saline pre-treated animals were available for immunohistochemistry. All animals in this group were anaesthetized with isoflurane and sacrificed using either cardiac perfusion or cervical dislocation under anaesthesia.
2. The autoradiography and HPLC experiments were approved by the Comité d’Ethique NOrmandie en Matière d’EXpérimentation Animale. For the autoradiography experiments N = 12 saline pre-treated and N = 12 AMPH pre-treated animals were included, whereas for the HPLC experiments N = 12 saline pre-treated and N = 11 AMPH pre-treated animals were used. All animals were sedated with isoflurane and euthanized by cervical dislocation under anaesthesia.
3. The storage phosphor imaging experiments were approved by the Animal Experiments Committee (DEC) at the Academic Medical Centre Amsterdam. For these experiments rats (N = 10 AMPH pre-treated, N = 10 saline pre-treated) were anesthetized with ketamine/xylazine mix and sacrificed using cardiac puncture under anaesthesia.

### phMRI for DA functionality

The phMRI experiments were carried out following a washout period of 7 days to ensure total AMPH clearance. During the MRI experiment, half of the rats in each experimental group received 4 mg/kg methylphenidate (MPH) i.p. (Sigma Aldrich, UK) dissolved in 0.9% saline in a volume of 0.3 ml to challenge the DA system. The other half of the animals received a saline challenge (same volume). This resulted in 4 experimental groups for the phMRI: pre-treated saline with saline challenge (SAL-SAL, N = 11), pre-treated saline with MPH challenge (SAL-MPH, N = 10), pre-treated AMPH with saline challenge (AMPH-SAL, N = 10) and pre-treated AMPH and challenge with MPH (AMPH-MPH, N = 11).

MRI experiments were performed using a 4.7 T Direct Drive Agilent (previously Varian, Palo Alto, CA) MRI system. Animals were placed in a linear radiofrequency coil with a volume with 72 mm inner diameter (m2m Imaging Corp., Cleveland OH, USA), which was used as a transmitter. The MR signal was received by four phased array coils (m2m Imaging Corp., Cleveland OH, USA) placed around the head of the animal. During the MRI scan, anaesthesia was maintained with 1.5–2.0% isoflurane and animals were ventilated in a 70:30 mixture of medical air and oxygen. Ventilation parameters, body temperature and heart rate were monitored throughout the scan.

For each animal, a T2 weighted anatomical image volume was acquired using a fast spin echo multi-slice sequence (fsems) with an echo train length of 8, matrix size = 256x256, FOV = 35x35 mm, 24 contiguous interleaved 1 mm coronal slices, 4 averages, 2 dummy scans, effective repetition time (TReff) = 5112 ms, and effective echo time (TEeff) = 60 ms. The time series were acquired using a gradient echo multi-slice (gems) sequence with 16 contiguous interleaved 1 mm slices centred to the same position as the anatomical image with TR = 260 ms, TE = 14 ms, flip angle 40 deg, 2 averages, 2 dummy scans, FOV = 35x35 mm and matrix size of 128x96 (zero-fill to 128x128), covering the regions of interest. Fifty time points (acquisition time per time series volume was 50 s; total scan time of approximately 41 minutes) were acquired with an injection of the pharmacological challenge after acquisition of volume 12.

### MRI data analysis

As a first step, the anatomical and time series data were converted to 4D Analyze format using ImageJ [14]. For image processing, the pixel dimensions were scaled by a factor of 10 to ensure compatibility with analysis algorithms designed for human data. This resulted in a voxel size of 2.73 × 2.73 × 10 mm^3^ for the time series data. Pre-processing included motion correction, which was applied by re-aligning the functional data to the first dynamic volume. In addition, anatomical and time series data were registered to a stereotactic rat brain template [15] using Statistical Parametric Mapping (SPM) software. Then, we co-registered the structural data to the rat template with 3, 6 and 12 degrees of freedom, respectively. Subsequently the data were normalized using a non-linear frequency cut-off of 15Hz. Next, all transformations were applied to the functional data [16]. Thereafter, the functional data were smoothed with a 3x3x7mm Gaussian kernel. Finally, brain extraction was obtained by multiplying a binary mask from the rat template with the functional data.

#### ROI-based analysis

We hypothesized the BOLD signal in certain DA-rich areas to be different between groups and therefore carried out ROI analyses in the CPu and NAcc. Mean time series per group per ROI were extracted from unsmoothed BOLD time series using a 3D digital reconstruction of a rat brain atlas [17] co-registered with the anatomical MRI template [15], using IDL-based software (Research Systems Inc., Boulder, Colorado). The anatomical definitions of the ROIs can be found in Gozzi et al. [18]. Statistical significance was assessed using SPSS version 20 (IBM, Chicago). We conducted a repeated-measures analysis with time (baseline (timepoint 1–12) and post-challenge (timepoint 15–35) as within-subject factor within each group to assess the change in BOLD signal compared to baseline. In addition, a repeated-measures analysis was done with time as within-subjects factor and group as between-subject factor to assess differences between groups. AMPH-MPH and SAL-MPH groups were compared with the two saline challenge groups combined (AMPH-SAL + SAL-SAL). In addition, the change in AMPH-MPH was compared to the SAL-MPH group to directly contrast the effect of pre-treatment on the response to MPH.

#### Exploratory voxel-based analysis

In addition to ROI-based analysis, exploratory whole brain analyses were conducted to explore the effects in regions downstream from DA projections. Image-based time series analysis was performed using *FEAT v*. *5*.*98*, part of *FSL* [19]. First level analysis was conducted using a model based on exploratory data analysis to obtain the shape of the hemodynamic response according to Klomp et al. [20]. In brief, Stimulate software [21] was used to extract changes in signal intensity after the MPH challenge. All individual time courses were then averaged to obtain the basic shape of the first level model, which was normalized and smoothed before entering into the design matrix. The design matrix was composed of this model and its temporal derivative. Higher-level mixed effect analysis was carried out using ordinary least squares simple mixed effects as implemented in *FSL FEAT* to determine group differences. Z (Gaussianised T/F) statistic images were thresholded using clusters determined by Z>1.6 and a (corrected) cluster significance threshold of p = 0.05 [22] (please see Z>1.9 and Z>2.3 thresholded maps in S1 Fig). AMPH-MPH and SAL-MPH groups were compared with the two saline challenge groups combined and were additionally tested against each other. The Paxinos and Watson rat brain atlas (1986) was used to identify location of significantly activated brain regions. Using the average waveform from the current data set as time regressor might have introduced a bias in the results, even though the waveform was heavily smoothed. To assess whether this was the case, we also conducted the analysis with a boxcar waveform. In order to capture a possible time lag of the MPH effects we also analysed a delayed boxcar time course [23]. These results are shown in S2 Fig, and are comparable to the initial waveform used. It shows that the onset of the effect is rather rapid, even though delayed effects are also found.

### Immunocytochemistry for GFAP, DAT, DRD1 and DRD2 assessments

Following MRI, a subset of animals (N = 15 AMPH pre-treated, N = 14 saline pre-treated) was perfused intracardially with saline followed by 4% paraformaldehyde in 0.1 M phosphate buffer (PB). To prevent pressure artefacts, brains were additionally post-fixed overnight in the skull at 4°C. The fixed brains were then saturated in a solution of 15% sucrose in PB (PB, 0.1M, pH 7.4) followed by 30% sucrose in PB for cryoprotection after which they were frozen and coronally sectioned in a one-in-ten series at 30 μm on a sledge microtome (Jung AG, Heidelberg, Germany). Immunocytochemistry was performed in the CPu and NAcc for: DAT (polyclonal rabbit anti DAT 1:2000, Novus Biologicals NBP1-19013), DRD1 (monoclonal mouse anti-DA receptor D1a 1:2000, Millipore MAB5290), DRD2 (polyclonal rabbit anti DA receptor D2a 1:400, Millipore AB5084P), and glial fibrillary acidic protein (GFAP, polyclonal rabbit anti-GFAP 1:2000, Dako Z0334) as described in detail in the S1 Supplementary Methods. Optical density was measured with the intensity function in *ImageJ* (Fiji, Image J) in one or multiple fixed-size regions. All sections were stained simultaneously and digitized with fixed settings. Light and background corrections were performed for all stainings except GFAP, due to the widespread distribution of GFAP.

### *In vitro* autoradiography for DAT and DRD1 assessments

Animals (N = 12 AMPH pre-treated, N = 12 saline pre-treated) were sedated with isoflurane and euthanized by cervical dislocation and the brains were rapidly removed, snap frozen and stored at -80°C. Frontal brain sections (14 μm) were cut and DAT and DRD1 autoradiography was performed on the CPu and NAcc. [^3^H]WIN35428 (Perkin-Elmer^®^, France; specific radioactivity = 3.034 MBq/nmol; 5 concentrations from 0.55 to 15.0 nM) was used for the DAT binding experiments according to protocols described before by Hebert [24]. Non-specific binding was determined by incubation of adjacent brain slices in the presence of 10 μM nomifensine. For the DRD1 binding experiments, [^3^H]SCH-23,390 (Perkin-Elmer^®^, France; specific radioactivity = 3.119 MBq/nmol; 5 concentrations from 0.10 to 8.1 nM) was used and performed according to the Savasta protocol [25]. Non-specific binding was determined by incubation of adjacent brain slices in the same conditions and in the presence of 10 μM SKF38393. Brain sections were exposed to tritium-sensitive phosphor imaging plates (Perkin-Elmer^®^) before acquisition of images (Cyclone^®^, Perkin-Elmer^®^). Specific binding was calculated as the difference between total and non-specific binding and K_d_ and B_max_ values were derived from raw data using nonlinear fitting procedures (Prism^®^).

### *Ex vivo* storage phosphor imaging for DRD2/3 assessments

Seven days following treatment, rats (N = 10 AMPH pre-treated, N = 10 saline pre-treated) were anesthetized with ketamine/xylazine mix followed by intravenous administration of approximately 50 MBq of the selective DRD2/3 tracer [^123^I]IBZM (GE Healthcare, Eindhoven, the Netherlands) into the tail vein. Ninety minutes later, rats were sacrificed using cardiac puncture under anaesthesia, and the brain was removed and frozen in nitrogen, sliced into horizontal slices of 50 μm using a microtome cryostat at -21°C. Storage phosphor imaging was performed as described previously [26]. In brief, every fifth slice was mounted on a glass plate and exposed to phosphor plates (Fuji BAS-MS IP) for the duration of 12 hours, allowing the phosphor plates to absorb energy emitted by radioactive decay from [^123^I]IBZM. The resulting luminescence emitted by the phosphor plates was scanned using a storage phosphor imager (GE Healthcare Typhoon FLA 7000) at a resolution of 25 μm using a 16-bit pixel depth, and analysed using *ImageQuant TL Toolbox* version 8.1. Regions of interest (ROIs) were the left and right CPu and the left and right NAcc, both DA-rich brain structures. The cerebellum was used to assess non-specific binding, as the cerebellum contains a negligible DRD2/3 density [27]. Specific dorsal CPu-to-cerebellum and NAcc-to-cerebellum ratios were obtained by dividing the average uptake per pixel of combined left and right CPu/NAcc parts by the average uptake per pixel of the cerebellum.

### *Ex vivo* HPLC for monoamine levels and metabolites

Brains (N = 11 AMPH pre-treated, N = 12 saline pre-treated) were rapidly dissected on cold plates; CPu and frontal cortex (FC) were dissected, weighed and stored at -80°C for further analysis. The tissue samples were homogenized for 30 min in 100 μl of an extraction solution (pH = 3) constituted of the mobile phase supplemented with perchloric acid 0.1 M. The mobile phase (MD-3MA, Thermo Scientific, France) was pumped at 0.4 mL/min with an isocratic high-performance liquid chromatography (UltiMate 3000 system, Thermo Scientific Dionex, France). Electrochemical detection (Coulochem III, Thermo Scientific Dionex, France) enabled the detection of monoamines and their metabolites. Peak quantification was determined using the Chromeleon 7.2 software (Thermo Scientific Dionex, France) and their concentrations derived from external standard curves. Concentrations of each compound were computed as the average of the two extracted values per sample.

### Statistics

Sample sizes were based on a previous study [13] with the same dosing regimen, taking into account possible drop-out due to complications of the treatment and/or MRI data quality. Power calculations showed that at least 10 rats per group were needed to detect an effect size of 1.8 (Cohen’s *d*). Rats were randomly assigned to either saline or AMPH treatment per cage and blinding was used for final statistical analyses. Data were analysed using two-sided independent t-tests or ANOVA to test for the effects of (i) repeated AMPH administration (ii) acute effect of MPH challenge. Data were assessed for normality using the Shapiro-Wilk test and equality of variance using Levene’s test. In case assumptions were violated, non-parametric tests were used. Corrections for multiple comparisons (NAcc and CPu) were conducted using the Benjamini-Hochberg procedure. Statistical analyses were performed using SPSS version 20.0 (IBM, Chicago) unless otherwise stated.

## Results

### phMRI for DA-ergic functionality

**Fig 1:**
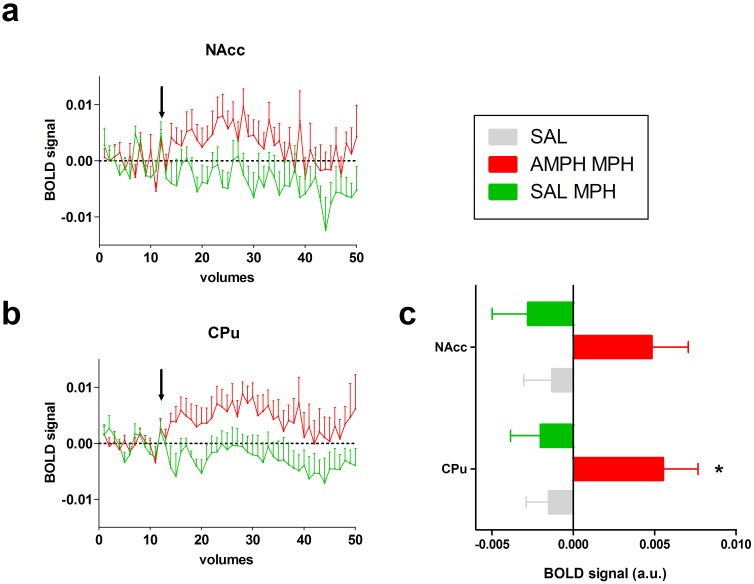
ROI analyses of phMRI data. phMRI time courses in the CPu (a) and NAcc (b). MPH or saline challenge was administered after 12 volumes (indicated by the arrow) followed by 38 volumes post-administration. The AMPH-MPH group differed significantly from the SAL-MPH and SAL groups. Difference between pre-injection (1–12) and post-injection (15–35) displayed for the CPU and Nacc (c) for each group (mean ±SEM)* p<0.05.

**Fig 2:**
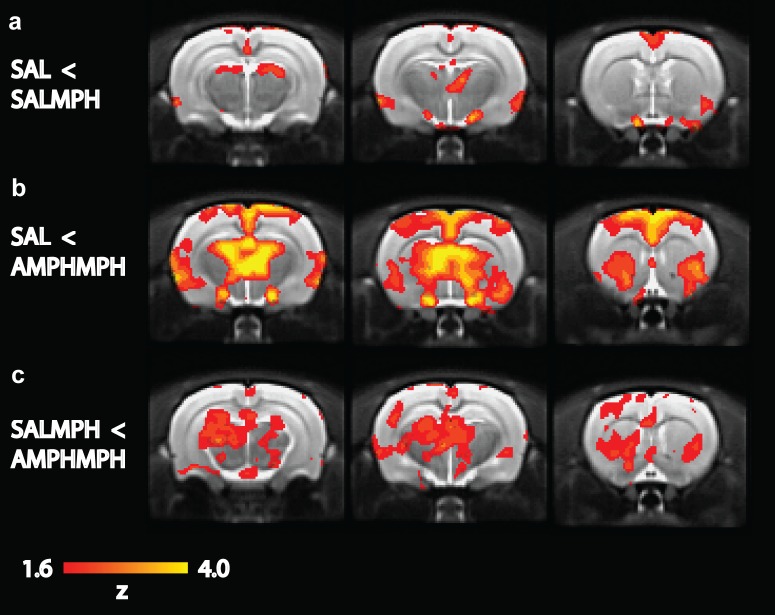
Whole brain analyses of phMRI data. The three rows show 1 mm-thick coronal slices for different group comparisons: a) MPH challenge in saline-treated rats increased BOLD response compared to groups that received a saline challenge b) MPH challenge in AMPH-pre-treated rats increased the BOLD response extensively c) AMPH-pre-treated rats show increased BOLD response compared to the SALMPH group. Images are thresholded at Z = 1.6.

The acute MPH challenge induced a significant BOLD response at post-challenge relative to baseline in the AMPH-MPH group in the CPu (CPu F_1,10_ = 6.60, p = 0.03; NAcc F_1,10_ = 4.64, p = 0.06; not significant after Benjamini-Hochberg correction), but not the SAL-MPH (CPu F_1,9_ = 1.20, p = 0.30; NAcc F_1,9_ = 1.60, p = 0.24) and SAL challenge groups (CPu F_1,20_ = 1.09, p = 0.31; NAcc F_1,20_ = 0.53, p = 0.47) (Fig 1). In addition, the change in BOLD response was higher in the AMPH-MPH group compared to the SAL groups combined (SAL-SAL + AMPH-SAL) in the CPu and NAcc (CPu F_1,30_ = 7.95, p<0.01; NAcc F_1,30_ = 4.40, p = 0.04), but not in the SAL-MPH group compared to the SAL groups combined (CPu F_1,29_ = 0.06, p = 0.82; NAcc F_1,29_ = 0.25, p = 0.62). When comparing the AMPH-MPH and SAL-MPH group directly, the AMPH-MPH group showed a significantly larger BOLD response than the SAL-MPH group (CPu F_1,19_ = 6.94, p = 0.02; NAcc F_1,19_ = 5.85, p = 0.03). The whole brain analyses were in agreement with the ROI analysis, demonstrating that an acute MPH challenge significantly activated a number of clusters of voxels in the thalamus, hippocampus (HC), CPu and cortically in fronto-temporal areas in the AMPH-MPH group (Fig 2) when compared to the vehicle groups together. In addition, the SAL-MPH group also showed small increases in BOLD signal compared to baseline in thalamus and temporal cortex.

The acute MPH challenge only induced positive BOLD responses, but the extent of activation was much larger in the AMPH-MPH group than in the SAL-MPH group. In the SAL-MPH group, we observed small bilateral increases in activation in the thalamus and temporal cortex. In contrast, in the AMPH pre-treated group, a large number of regions showed a bilateral increase in BOLD signal intensity, including the thalamus, HC, CPu and cortically in fronto-temporal areas. When the SAL-MPH and AMPH-MPH groups were compared directly, we found the strongest group differences in subcortical DA-rich structures, such as the striatum, thalamus and substantia nigra.

### Immunocytochemistry for GFAP, DAT, DRD1 and DRD2 assessments

**Fig 3:**
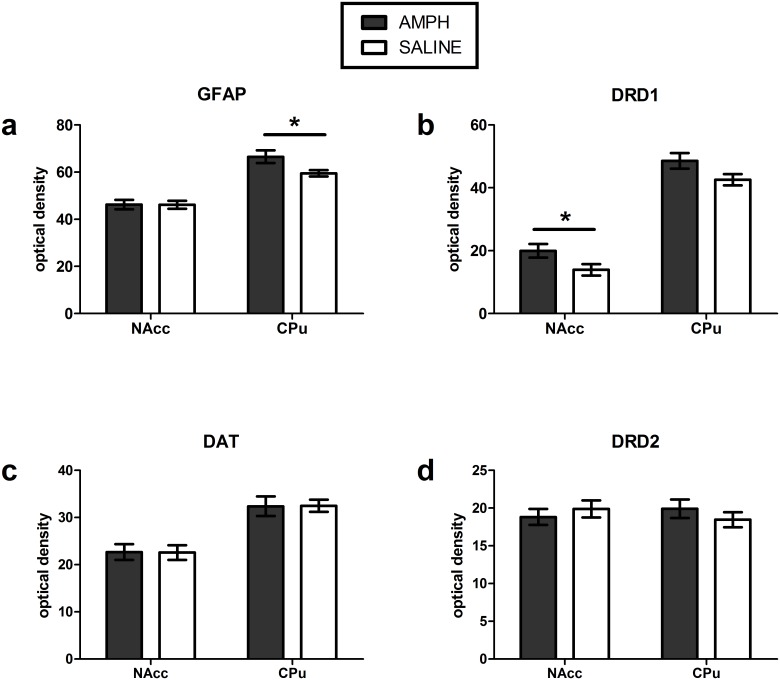
Immunocytochemistry. a) GFAP b) DRD1 c) DAT and d) DRD2 expression following AMPH or saline pre-treatment in the NAcc and CPu (mean ±SEM) * p<0.05.

AMPH pre-treatment, compared to saline pre-treatment, resulted non-significant higher DRD1 immunoreactivity in the CPu (+14.1%, t_(27)_ = 1.95, p = 0.06) and significantly higher expression in the NAcc (+43,3%, t_(27)_ = 2.14, p = 0.04; not significant after Benjamini-Hochberg correction), as well as higher GFAP immunoreactivity in the CPu (+11.8%, U = 59 p = 0.046 not significant after Benjamini-Hochberg correction) (Fig 3 and S3 and S4 Figs). No differences were found in DRD1 or GFAP expression in other ROIs. AMPH pre-treatment did not significantly affect DRD2 or DAT immunoreactivity when compared to saline pre-treatment (S5 and S6 Figs).

### *In vitro* autoradiography for DAT and DRD1 assessments

AMPH pre-treatment resulted in a significant 21–24% lower binding of DAT in the CPu (t_(18)_ = 5.56, p<0.001), NAcc (t_(21)_ = 9.26, p<0.001) compared to saline pre-treatment. DRD1 density was significantly lower in the NAcc (-7% t_(22)_ = 2.83, p<0.01) but not altered in the CPu (t_(22)_ = 0.78, p = 0.40). Mean and standard error of the mean (SEM) are displayed in Table 1 (S7 Fig illustrates the distribution of DAT and DRD1 binding sites).

**Table 1:** Effect of pre-treatment with AMPH on DAT and DRD1 binding (autoradiography).

	DAT [3H]-WIN35428 B_max_ fmol/mg TE, mean ± SEM		DRD1 [3H]-SCH23390 B_max_ fmol/mg TE, mean ± SEM	
	Saline	AMPH	Saline	AMPH
**CPu**	192 ± 5	151 ± 4***	361 ± 7	353 ± 8
**NAcc**	407 ±9	308 ± 5***	360 ± 5	335 ± 7**

** p<0.01 *** p<0.001 (AMPH vs saline)

### *Ex vivo* storage phosphor imaging for DRD2/3 assessments

There was no significant difference in DRD2/3 receptor availability in the NAcc (t_(18)_ = 1.45, p = 0.16) or the CPu (t_(18)_ = 1.05, p = 0.31) between groups pre-treated with saline versus AMPH. Representative phosphor images from the AMPH and saline group are displayed in S8 Fig, as well as the mean and SEM.

### *Ex vivo* HPLC for monoamine levels and metabolites

AMPH pre-treatment did not result in significantly different monoamine levels, except for increases in HVA levels in the CPu (+231%, p<0.0001) (Table 2). No differences were found in the frontal cortex.

**Table 2:** Effect of pre-treatment with AMPH on brain monoamines and metabolites.

	NA	MHPG	DA	DOPAC	HVA	5-HT	5-HIAA
**CPu**							
**Saline**	0.149 ± 0.022	*ND*	2.162 ± 0.244	1.923 ± 0.199	1.295 ± 0.195	0.217 ± 0.014	0.305 ± 0.022
**AMPH**	0.162 ± 0.030	*ND*	2.001 ± 0.273	2.302 ± 0.268	4.293 ± 0.538****^a^	0.227 ± 0.026	0.246 ± 0.028
**Cortex**							
**Saline**	3.390 ± 1.104	3.884 ± 0.580	0.043 ± 0.005	0.008 ± 0.001	0.378 ± 0.195	0.497 ± 0.026	0.369 ± 0.023
**AMPH**	2.950 ± 0.261	3.212 ± 0.285	0.053 ± 0.018	0.006 ± 0.001	0.381 ± 0.050	0.478 ± 0.029	0.342 ± 0.019

Data (mean ± SEM) are concentrations (μg/g wet tissue) of monoamines (NA: Noradrenaline, DA: Dopamine, 5-HT: Serotonin) and metabolites (MHPG: 3-methoxy-4-hydroxyphenylglycol, DOPAC: 3,4-dihydroxyphenylacetic acid, HVA: homovanilic acid, 5-HIAA: 5-hydroxyindolacetic acid) in the striatum in saline and AMPH pre-treated rats, one week after the treatment.

^a^ AMPH *vs*. Saline: *p<0*.*0001*;

ND Not Detected.

## Discussion

We here used phMRI to investigate in rats the consequences of repeated AMPH administration, known to damage the DA synapse. We assessed whether a subsequent MPH challenge would allow to detect changes in DAT, DA receptor, DA levels and its metabolites, and therefore also validated these measures by analyzing them post-mortem. We demonstrate that AMPH pre-treatment increases the BOLD response to an acute MPH challenge in DA-innervated brain regions. The changes in BOLD response possibly reflect increased DA-ergic post-synaptic stimulation as supported by the higher HVA and reduced striatal DAT binding we found. However, based on autoradiography and ICC, contrasting results were found for DRD1 expression following AMPH treatment.

### Repeated AMPH treatment induces exacerbated BOLD response

In accordance with Belcher et al. [13], our AMPH pre-treatment model induced reduction in DAT binding. In addition, the AMPH pre-treatment induced a higher BOLD response to an acute MPH challenge in subcortical DA areas. This exacerbated DA-ergic response to MPH may either be due to an increase in DA release and/or a reduction in its re-uptake or metabolism. This would result in the presence of more DA in the synaptic cleft and hence an increase in post-synaptic neurotransmission in AMPH pre-treated rats. Enhanced striatal DA release has been shown to induce both cortical and subcortical hemodynamic responses, reflecting its arousing and rewarding properties [28].

When animals are repeatedly exposed to psychostimulant drugs, the re-administration of the drug after a period of withdrawal results in an exaggerated DA release, which is consistent with the exacerbated BOLD response we observed following the MPH challenge. Additionally, the presently observed reduction in DAT binding could result in a decreased re-uptake and therefore elevated DA levels, possibly further augmenting DA neurotransmission [7]. Interestingly, these findings differ from clinical studies from our group, in which we found a blunted phMRI response and striatal DA release in regular AMPH users [29,30]. Yet, Boileau et al. (2006) found sensitization in humans who repeatedly received AMPH under similar conditions. This discrepancy between rodent and human studies is also observed for the effects of cocaine [31] and may be due to differences in timing and duration of administration (i.e. at an early stage of drug abuse vs. dependence, or after a wash-out period) as well as to effects of the environmental context, i.e. in relation to reward anticipation. Alternatively, the discrepancy between human and animal studies could possibly be explained by the use of anaesthesia. Although we cannot rule out the possibility that isoflurane has influenced our results (there has been some discussion on the effect of isoflurane on the DA system [32,33]), all groups received the same level of anaesthesia, so this is unlikely this explains the large difference between our AMPH and saline groups. Previous studies had further suggested, that similar levels of anaesthesia, as in the present study, do not affect the sign and distribution of phMRI responses [34]. Future experiments in awake rats could advance the comparability between animal and human studies.

PhMRI studies reported in the literature have demonstrated that DA-ergic stimulants (including AMPH and MPH) elicit significant changes in the hemodynamic response of DA-innervated brain regions of healthy animals [8,10]. Yet in the saline pre-treated rats, we observed a small BOLD response as compared to AMPH-pretreated rats. It has been demonstrated previously that low doses of AMPH can induce a small hemodynamic response, probably due to a higher affinity for DRD2 (auto)receptors. These autoreceptors are expressed on the pre-synaptic terminal and their stimulation results in a lower DA release. Alternatively, higher doses of AMPH increase the phMRI signal more as a result of an increased binding of DA to DRD1 [35]. The current literature is somewhat equivocal, with both increases and decreases of the phMRI signal being reported [36–39] which may relate to methodological differences, such as the route and dose of MPH administration as well as the ventilation protocol used.

### What neurobiological changes drive the phMRI response?

We here observed that AMPH treatment reduced DAT binding significantly in the CPu (-21%) and NAcc (-24%) as measured with [^3^H]WIN-35428 autoradiography. At first glance, this does not appear in concordance with our ICC findings where an no change in DAT was apparent. However, this can be explained by the fact that ICC measures the entire pool of DAT (i.e. intra- and extracellular, functional and not functional), whereas autoradiography assesses only the functional membrane DAT to which ligand can bind. Hence, the autoradiography data suggest that the active pool of DAT, and/or their activity, is reduced in AMPH-treated rats, corroborating our hypothesis of DAT-dependent changes in phMRI signal, whereas the entire pool (as measured with ICC) may have been increased as a compensatory response. Similarly, our results in DRD1 expression differ when measured with autoradiography and ICC. This suggests that although there might be an increase in the overall pool of DRD1 receptors, fewer receptors are functionally available (as supported by the autoradiography data), possibly due to an exaggerated DA neurotransmission. However, the reason for the increase in overall DRD1 levels remains elusive and requires further research. The concept of an AMPH-induced disruption of normal DA-ergic functioning is further supported by the increases in GFAP, a classical marker of astrogliosis, as has been reported before under similar, as well as degenerative conditions [40].

Furthermore, levels of the DA metabolite HVA were elevated in AMPH-pretreated rats while no differences were present in basal DA levels. Both DA and HVA were found to be increased following acute AMPH [41], likely as a result of reversed DA transport [40] or internalization of DAT [42], that would lead to an increase in the amount of DA in the synaptic cleft that can be metabolized. Moreover, the decreased DAT binding can result in less reuptake and more available DA to be metabolized [43]. In addition, acute AMPH has been shown to inhibit the activity of the enzyme monoamine oxidase (MAO) [40,44], initially resulting in reduced DA metabolism. One explanation could therefore be that, following one-week of washout and disinhibition of MAO, DA metabolism is still increased, resulting in higher HVA levels. Alternatively, our phMRI data suggest a slower breakdown of the released DA due to deficient DAT reuptake, which might again increase DA metabolism and thus result in higher HVA levels. Interestingly, no changes in HVA were found in the frontal cortex, a region with lower DAT expression than the CPu, where catechol-O-methyltransferase (COMT) and MAO are thought to play a larger role in the modulation of DA neurotransmission [44]. This suggests that AMPH-induced DAT deficiency has a larger impact on changes in DA metabolism in the CPu than in the frontal cortex.

### Methodological considerations

This experiment used BOLD contrast to visualize hemodynamic changes, instead of contrast-enhanced cerebral blood volume (CBV) phMRI studies. Although CBV-weighted imaging might provide a higher sensitivity, BOLD contrast is more often used in the clinical research setting and our findings can therefore be more easily translated to the human situation. Systemically administered challenges can induce systemic changes to various physiological parameters that can hamper interpretation of the BOLD response. For these reasons, blood pressure and pCO_2_ levels were carefully monitored in our study while previous studies have shown that systemic changes as a result of i.v. administration with AMPH did not affect phMRI results [8]. We used i.p. administration of MPH, which will induce less systemic changes and these are therefore considered negligible for the interpretation of our results. Although we cannot exclude some bias in the waveform applied for the whole-brain analyses, using box car regressors provided us with similar results, therefore making the possibility of bias less likely.

Finally, it is important to note that psychostimulants such as AMPH and MPH not only affect the DA system, but also act on the noradrenergic (NA) system and can have downstream effects on other neurotransmitter systems. Especially in cortical areas, where NAergic neurons are involved in regulating DA [45], MPH-induced changes may, at least in theory, involve mixed DA-ergic and NAergic activation. Although we did not find any changes in NA (metabolites) in our AMPH treated rats, we cannot completely exclude this further research into this will be of interest.

### Conclusions

We report that AMPH pre-treatment, which is known to induce neurotoxic changes in the DA synapse, induced an exaggerated phMRI response to a subsequent DAergic challenge in rats. Our validation data indicate that the phMRI signal changes are likely explained by increases in DA neurotransmission. Consistent with literature, we demonstrate that repeated AMPH reduced striatal DAT and increased HVA levels. This supports that phMRI is sensitive enough to measure complex alterations in the DA system. This ability to assess subtle synaptic changes is promising for both preclinical studies of DA-ergic drug discovery and monitoring as well as for future clinical studies where phMRI can be used to investigate DA abnormalities, e.g. in various neuropsychiatric disorders, in a non-invasive way.

## Supporting information

S1 Supplementary Methods (DOC)

S1 Supplementary Data (SAV)

S1 Fig Additional whole brain analyses with more stringent thresholds. The analyses were exactly the same as reported in the results section, but with higher z-thresholding. The top three rows are thresholded at z>1.9 and the bottom three rows are thresholded at z>2.3.
(TIF)

S2 Fig Additional whole brain analyses with boxcar regressors. Left) boxcar regressor with immediate response following MPH administration (EARLY); right) boxcar regressor with lagged response following MPH administration (after 29 volumes) (LATE); The images are thresholded at z>1.6.
(JPG)

S3 Fig DRD1 expression in the CPu and NAcc in representative rats for each group. (TIF)

S4 Fig GFAP expression in the CPu and NAcc in representative rats for each group. (TIF)

S5 Fig DAT expression in the CPu and NAcc in representative rats for each group. (TIF)

S6 Fig DRD2 expression in the CPu and NAcc in representative rats for each group. (TIF)

S7 Fig Representative illustration of the cerebral distribution of DAT (B) and DRD1 (C) binding sites atthe level of the CPu and NAcc (A; Bregma + 1.60 mm) in a control rat. (TIFF)

S8 Fig Storage phosphor imaging for DRD2/3 assessments. a) Examples of regions of interest for CPu (A), NAcc (B) and cerebellum (C). b) DRD2/3 binding potential in the CPu and NAcc in AMPH and saline pre-treated rats (mean+SEM).
(TIF)
