# Supplementary material for: Repeated dexamphetamine treatment alters the dopaminergic system and increases the phMRI response to methylphenidate
Source: PLoS One. 2017 Feb 27;12(2):e0172776. doi: 10.1371/journal.pone.0172776 (PMC5328278; doi:10.1371/journal.pone.0172776)
Supplement: S1 Supplementary Methods — (DOC) [file pone.0172776.s001.doc]

**Supplementary Methods**

*phMRI*

Following pre-treatment and a washout period of 5 to 7 days to ensure total drug clearance, rats were anaesthetised with isoflurane (5% induction and then reduced to 1.5-2% for maintenance of anaesthesia during animal preparation and scanning given in a 70:30 mixture of medical air and oxygen. The right femoral artery was cannulated for blood gas measurements (RapidLab 348, Siemens Healthcare Diagnostics, Newbury, UK) and blood pressure (Biopac Systems Corp., Goweta, USA) monitoring. Subsequently, the animal was tracheotomised and artificially ventilated with a mechanical respirator (Inspira ventilator, Harvard Apparatus). To assess whether artificial ventilation was successful, respiration was monitored using a respiratory cuff coupled to a pressure sensor (SA Instruments, New York, USA). Ventilation parameters were adjusted for each animal before the scan such that its blood gas values remain within the physiological range (else subjects were excluded from the analyses). No statistically significant difference in the pre- and post-administration pCO_2_ values between groups was found (ANOVA F(3,36)=1.8; p=0.17). Mean arterial blood pressure (MAP) was also closely monitored during the MRI experiment. Body temperature was monitored with a rectal probe and maintained at 37.5 ± 1 ºC by a warm air heating system (SA Instruments, New York, USA). Finally, an i.p. cannula was placed for delivery of the pharmacological challenge during the functional scan. During the MRI experiment, in each experimental group half of the rats received 4 mg/kg MPH i.p. (Sigma Aldrich, UK) dissolved in 0.9% saline in a volume of 0.3 ml to challenge the DA system. The other half received a saline challenge.

*Immunocytochemistry*

After sectioning, the sections were washed twice in phosphate buffer (PB) to get rid of remaining sucrose and were subsequently stored in PB-azide (0.1M phosphate buffer, pH7.4 + 0.01% sodium-azide) and stored at 4ºC until further use. To remove sodium azide, sections were washed three times with buffer (for staining-specific reagent details, see Supplementary Table 1). Endogenous peroxidase activity was blocked with 0.5% H_2_O_2_ in buffer solution, followed by four washes. Next, non-specific binding was prevented by incubation in protein blocking solution. The sections were then incubated with the primary antibody in primary incubation solution for 1 h at room temperature (RT) and overnight (ON) at 4°C. The following day, the sections were allowed to acclimatize to RT and were subsequently washed for five times. The sections were incubated for 2 h at RT with a biotinylated secondary antibody in the secondary incubation solution. Next, the sections were washed four times before the sections were incubated in Vectastain Elite ABC (PK-4000, Vector Labs) at 1:800 dilution for 2 h the sections were then washed once followed by three washes with Tris-HCl buffer (TB, 0.05M, pH7.6) The staining was visualized with 0.05% 3,3-“-Diaminobenzidine tetra-hydrochloride (DAB, Sigma, no D-5637) with 0.01% H_2_O_2_ in TB, whereas the staining reaction was stopped by several washes in TB. Finally, the sections were mounted with 0.2% gelatin in TB and air dried ON. A gentle haematoxylin counterstaining according to Ehrlich was performed on GFAP sections only. After dehydration with alcohol and clearing in xylene the sections were covered with Entallan.
 The following brain structures were located (caudate putamen, CPu, bregma 2.28; Nucleus Accumbens Core, NAcc, bregma 2.28) according to the rat brain atlas [1]. The brain areas were digitized with a Zeiss Axiophot microscope (Carl Zeiss Microscopy GmbH, Jena, Germany) equipped with a Optronics Microfire camera and StereoInvestigator software (MBF bioscience) at 2.5x magnification with optimal and fixed settings for each staining within a brain structure.

*Autoradiography*

Animals were euthanized by vertebral dislocation and the brains were rapidly removed, frozen in isopentane at 40°C and stored at -80°C. Coronal brain sections (14µm) were obtained with a cryomicrotome (Leica® CM3050) and mounted onto gelatin-coated slides. DAT binding experiments were performed according to Hebert et al. (1999). In brief, brain slices were pre-incubated for 20 min in 30 mM sodium phosphate buffer (pH 7.4, +4°C), and then incubated for 90 min in the presence of [^3^H]WIN35428 (Perkin-Elmer®, France; specific radioactivity = 3.034 MBq; 5 concentrations from 0.55 to 15.0 nM) in the same buffer supplemented by 0.32 mM sucrose. Non-specific binding was determined by incubation of adjacent brain slices in the presence of 10µM nomifensine. Brain slices were rinsed (3 x 1 min) in ice-cold sodium phosphate buffer (pH 7.4 at + 4°C), rapidly dipped in ice-cold distilled water and dried under a stream of cooled air. D1R binding experiments were performed according to Savasta et al. (1986). In brief, brain slices were pre-incubated for 15 min in 50 mM Tris-HCl buffer supplemented with 120 mM NaCl, 5 mM KCl, and 1 mM MgCl2 (pH 7.4, +25°C), and then incubated for 60 min in the presence of [^3^H]SCH-23,390 (Perkin-Elmer®, France; specific radioactivity = 3.119 MBq; 5 concentrations from 0.10 to 8.1 nM) in the same buffer in the presence of 30 nM ketanserin. Non-specific binding was determined by incubation of adjacent brain slices in the same conditions and in the presence of 10µM SKF38393. Brain slices were rinsed (2 x 1 min) in ice-cold 50 m MTris-HCl, 120 mM NaCl, 5 mM KCl, 1 mM MgCl2 buffer (pH 7.4 at + 4°C), rapidly dipped in ice-cold distilled water and dried under a stream of cooled air. Brain sections were co-exposed with standards ([^3^H] microscales, Amersham®) on tritium-sensitive phosphor imaging plates (Perkin-Elmer®) for ten days at room temperature before acquisition of images (Cyclone®, Perkin-Elmer®). Binding density of receptors (fmol/mg of tissue equivalent) was quantified with a computer-assisted image analyzer (OptiQuant®, Perkin-Elmer) using a three-order polynomial relation between optical densities and radioactivity. Specific binding was calculated as the difference between total and non-specific binding and K_d_ and B_max_ values were derived from raw data using nonlinear fitting procedures (Prism®). The same brain regions and bregmas were used as for the immunocytochemistry.

*HPLC*

Brains were rapidly dissected on a cold plate; CPu and frontal cortex were dissected, weighed and stored at -80°C for further analysis. The tissue samples were homogenized 30 min in 100 µl of an extraction solution (pH=3) constituted of the mobile phase supplemented with perchloric acid 0.1M. The samples were then centrifuged (10 000 rpm, 10 min), the supernatant were isolated and centrifuged again (10 000 rpm, 5 min). Each sample was injected two-times using a Rheodyne 7725i injector valve with a 20-µL injection loop. The mobile phase (MD-3MA, Thermo Scientific, France) was pumped at 0.4 mL/min with an isocratic high-performance liquid chromatography (UltiMate 3000 system, Thermo Scientific Dionex, France). Separation was performed with a 3-µm C18, 3.2 x 100 mm reversed phase column (MD-150, Thermo Scientific Dionex, France) maintained at 26°C. Electrochemical detection (Coulochem III, Thermo Scientific Dionex, France) using an analytical cell (5014B, E1 = -150 mV and E2 = +200 mV, Thermo Scientific Dionex, France) and a guard cell set at +350 mV (5020, Thermo Scientific Dionex, France) enabled the detection of monoamines and their metabolites. Peak quantification was determined using the Chromeleon 7.2 software (Thermo Scientific Dionex, France) and their concentrations derived from external standard curves. Concentrations of each compound were computed as the average of the two extracted values per sample. DA and its metabolites 3,4-Dihydroxyphenylacetic acid (DOPAC) and homovanillic acid (HVA) were detected in all the regions of interest. All the compounds were resolved in a 15 min run.

**References**

1. Paxinos G, Watson C. The rat brain in stereotactic coordinates. 5th ed. New York: Academic Press; 2005.

2. Hebert MA, Larson GA, Zahniser NR, Gerhardt GA. Age-Related Reductions in [3H]WIN 35,428 Binding to the Dopamine Transporter in Nigrostriatal and Mesolimbic Brain Regions of the Fischer 344 Rat. J Pharmacol Exp Ther. 1999;288: 1334–1339. Available: http://jpet.aspetjournals.org/content/288/3/1334.short

3. Savasta M, Dubois A, Scatton B. Autoradiographic localization of D1 dopamine receptors in the rat brain with [3H]SCH 23390. Brain Res. 1986;375: 291–301. Available: http://www.ncbi.nlm.nih.gov/pubmed/2942221

**Table A. Immunocytochemistry methods.**

|  | **GFAP** | **DRD1** | **DRD2** | **DAT** |
| --- | --- | --- | --- | --- |
| **Buffer solution** | 0.05M TBS pH7.6 | 0.05M PBS | 0.05M TBS | 0.05M PBS |
| **Protein block** | none | 3% Normal Goat Serum (NGS, life technologies, PCN5000 ) + 1% Bovine Serum Albumin ( BSA, Roche, Germany) | 3% NGS + 1% BSA + 0.2% Triton X-100 (merck, Germany) | 5% NGS + 0.1% Triton X-100 |
| **Incubationmix primary antibody** | 1% Milkpowder (Campina) + 1% Triton X-100 | 3% NGS + 1% BSA | 3% NGS + 1% BSA + 0.2% Triton X-100 | 0.25% gelatin (Merck, Germany) + 0.1% Triton X-100 |
| **Primary antibody** | polyclonal rabbit-anti-GFAP 1:2000 (DAKO, Z0334 ) 1hour at Room temperature, overnight at 4˚C | monoclonal mouse anti-dopamine D1a receptor 1:2000 (Millipore, MAB5290) | polyclonal rabbit-anti-Dopamin D2 receptor 1:400 (Millipore, AB5084P) | policlonal rabbit-anti-DAT 1:2000 (Novus, NBP1-19013) |
| **Incubationmix secondary antibody** | 1% triton X-100 + 1% BSA | 0.05M PBS | 0.05M TBS | 0.25% gelatin + 0.1% Triton X-100 |
| **Secondary antibody** | biotinylated goat-anti-rabbit IgG (Vector, 6-BA-1000) | biotynilated sheep-anti-mouse 1:200 (GE healthcare, RPN1001) | biotinylated goat-anti-rabbit IgG (Vector, 6-BA-1000) | biotynilated goat-anti-rabbit 1:200 (Vector, 6-BA-1000) |
| **ABC amplyfication** | 1:800 ABC-elite (Vector, PK-4000) + 1%BSA | 1:800 ABC-elite (Vector, PK-4000) | 1:800 ABC-elite (Vector, PK-4000) | 1:800 ABC-elite (Vector, PK-4000) |
| **Visualisation** | 0.5mg/ml 3,3-'-Diaminobenzidine tetra-hydrochloride (Sigma, Netherlands) +0.01% H_2_O_2_ | 0.5mg/ml 3,3-'-Diaminobenzidine tetra-hydrochloride + 0.01% H_2_O_2_ | 0.5mg/ml 3,3-'-Diaminobenzidine tetra-hydrochloride + 0.01% H_2_O_2_ | 0.5mg/ml 3,3-'-Diaminobenzidine tetra-hydrochloride + 0.01% H_2_O_2_ |
| **Haematoxalin counterstaining according to meyer** | 30 sec Haematoxilyn, 45 minutes running tapwater | none | None | none |
