# Supplementary figures and images for: Repeated dexamphetamine treatment alters the dopaminergic system and increases the phMRI response to methylphenidate
Source: PLoS One. 2017 Feb 27;12(2):e0172776. doi: 10.1371/journal.pone.0172776 (PMC5328278; doi:10.1371/journal.pone.0172776)

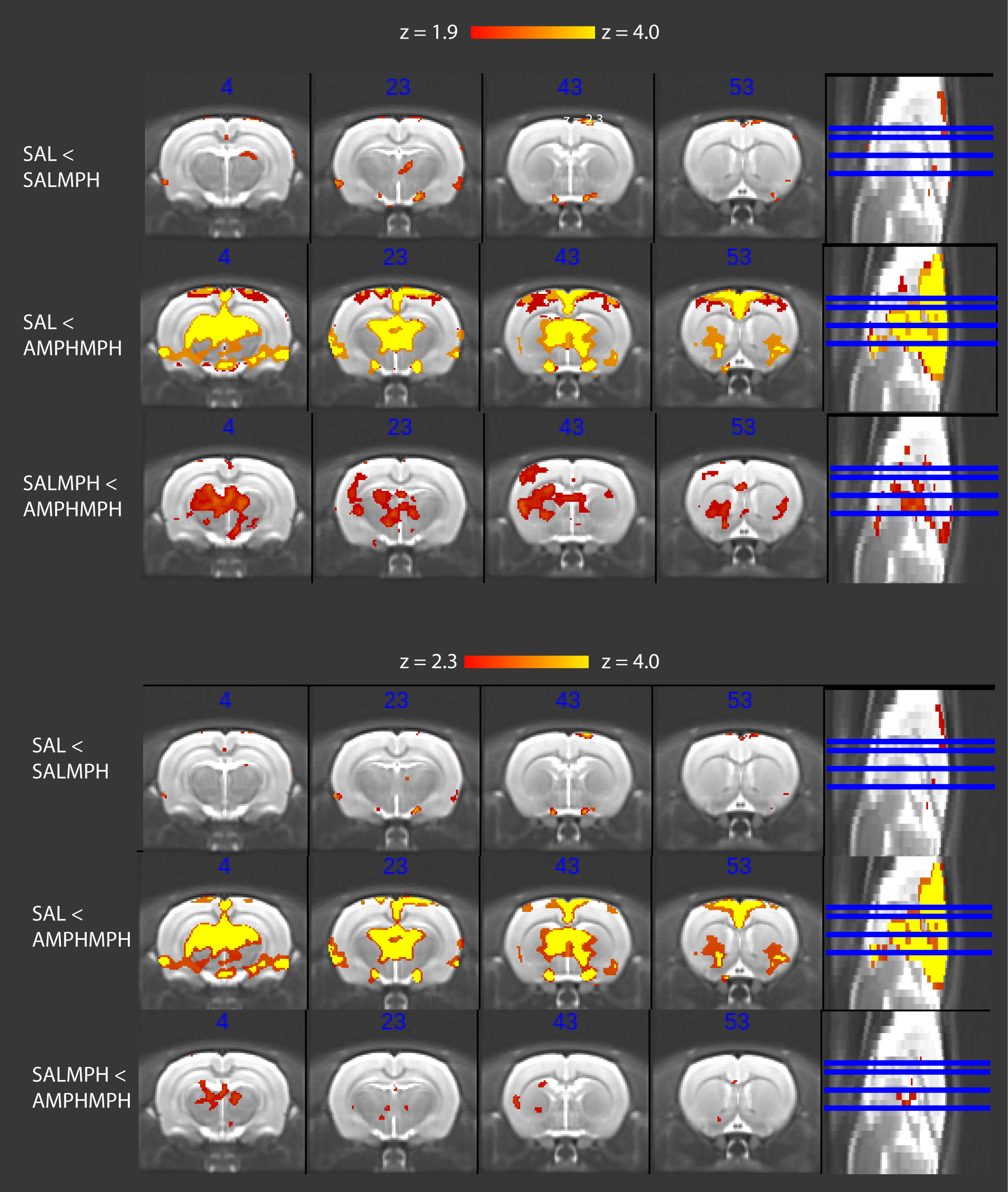

Supplement: S1 Fig — The analyses were exactly the same as reported in the results section, but with higher z-thresholding. The top three rows are thresholded at z>1.9 and the bottom three rows are thresholded at z>2.3. (TIF) [file pone.0172776.s003.tif]

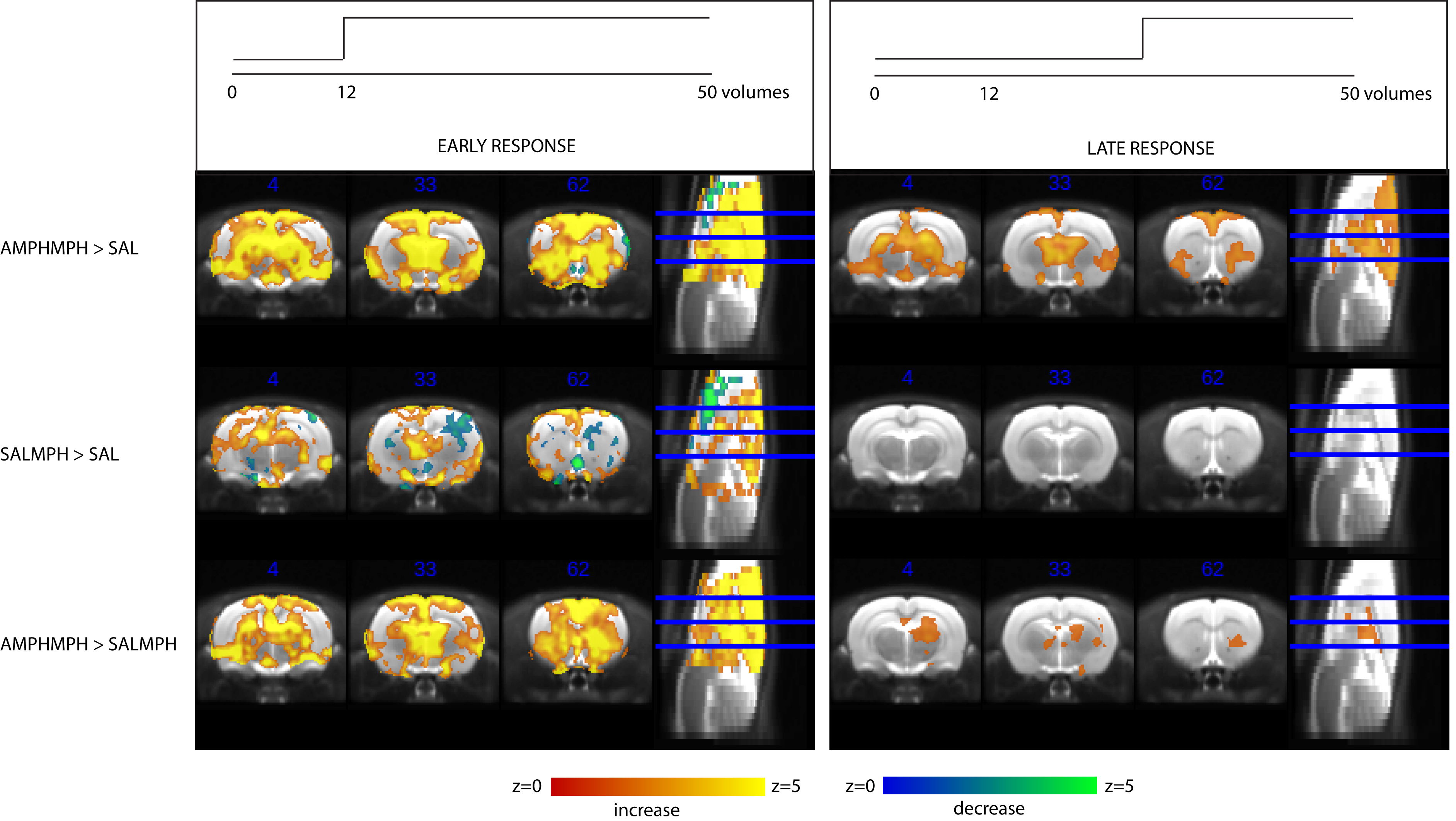

Supplement: S2 Fig — Left) boxcar regressor with immediate response following MPH administration (EARLY); right) boxcar regressor with lagged response following MPH administration (after 29 volumes) (LATE); The images are thresholded at z>1.6. (JPG) [file pone.0172776.s004.jpg]

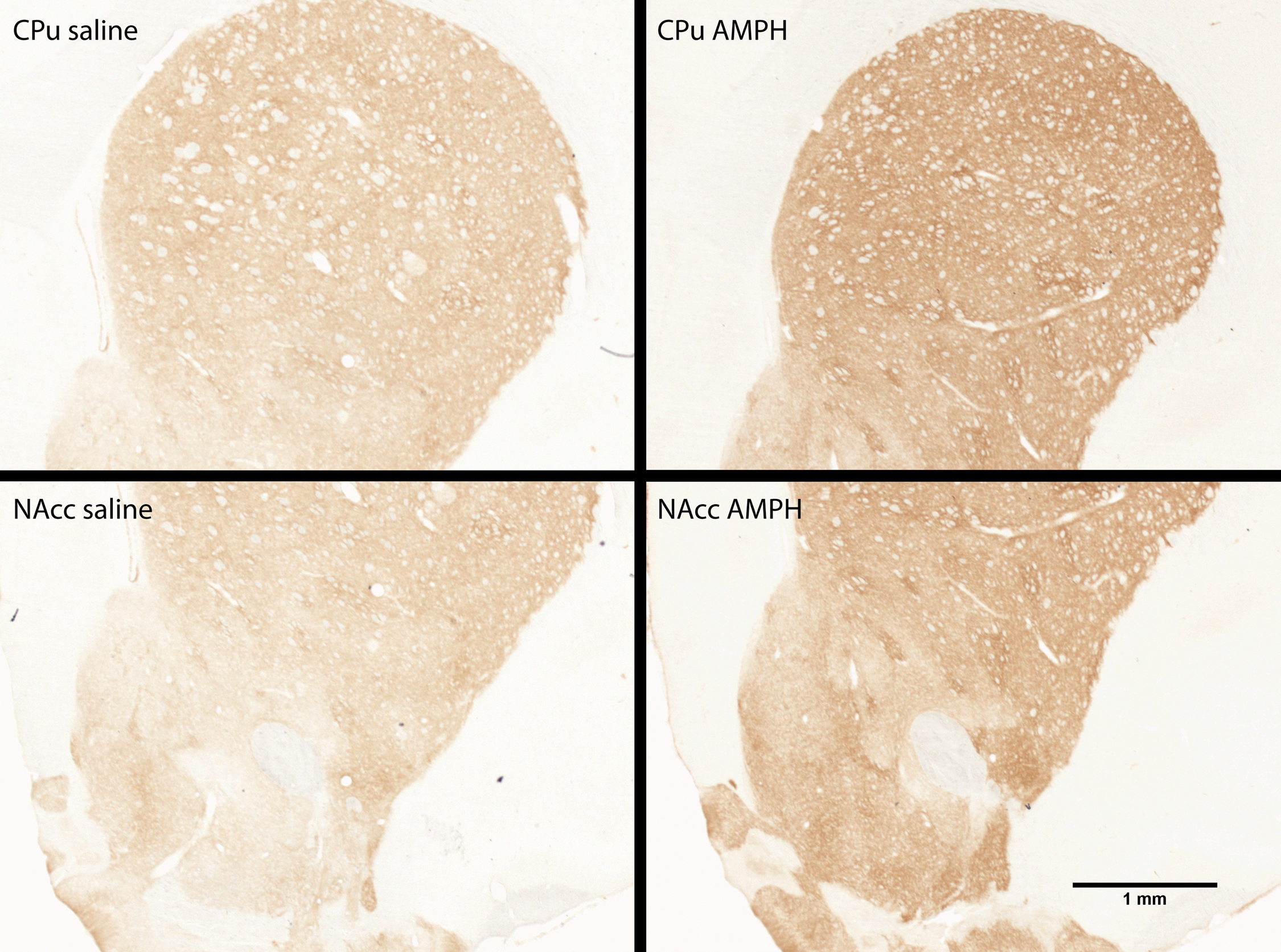

Supplement: S3 Fig — (TIF) [file pone.0172776.s005.tif]

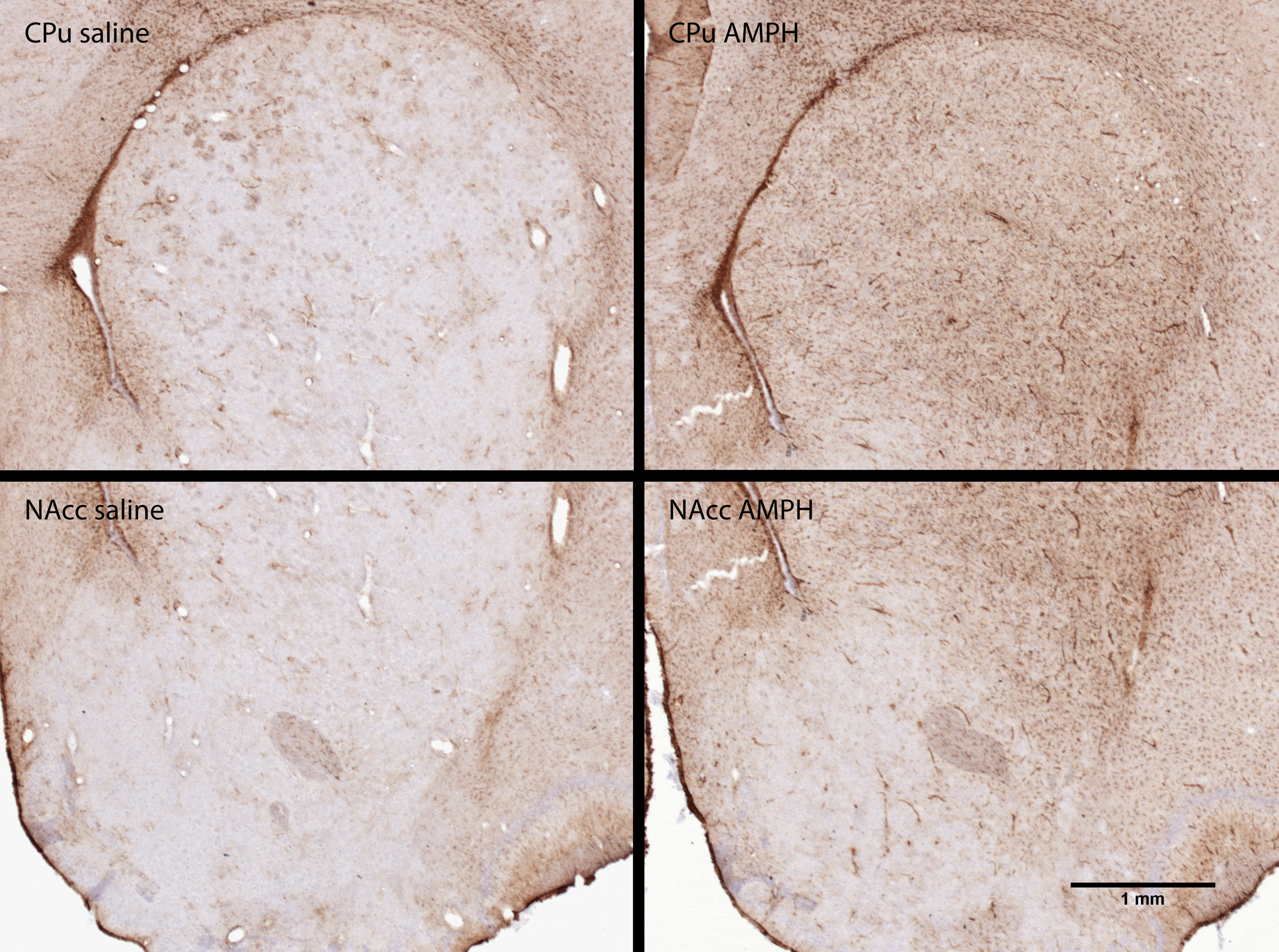

Supplement: S4 Fig — (TIF) [file pone.0172776.s006.tif]

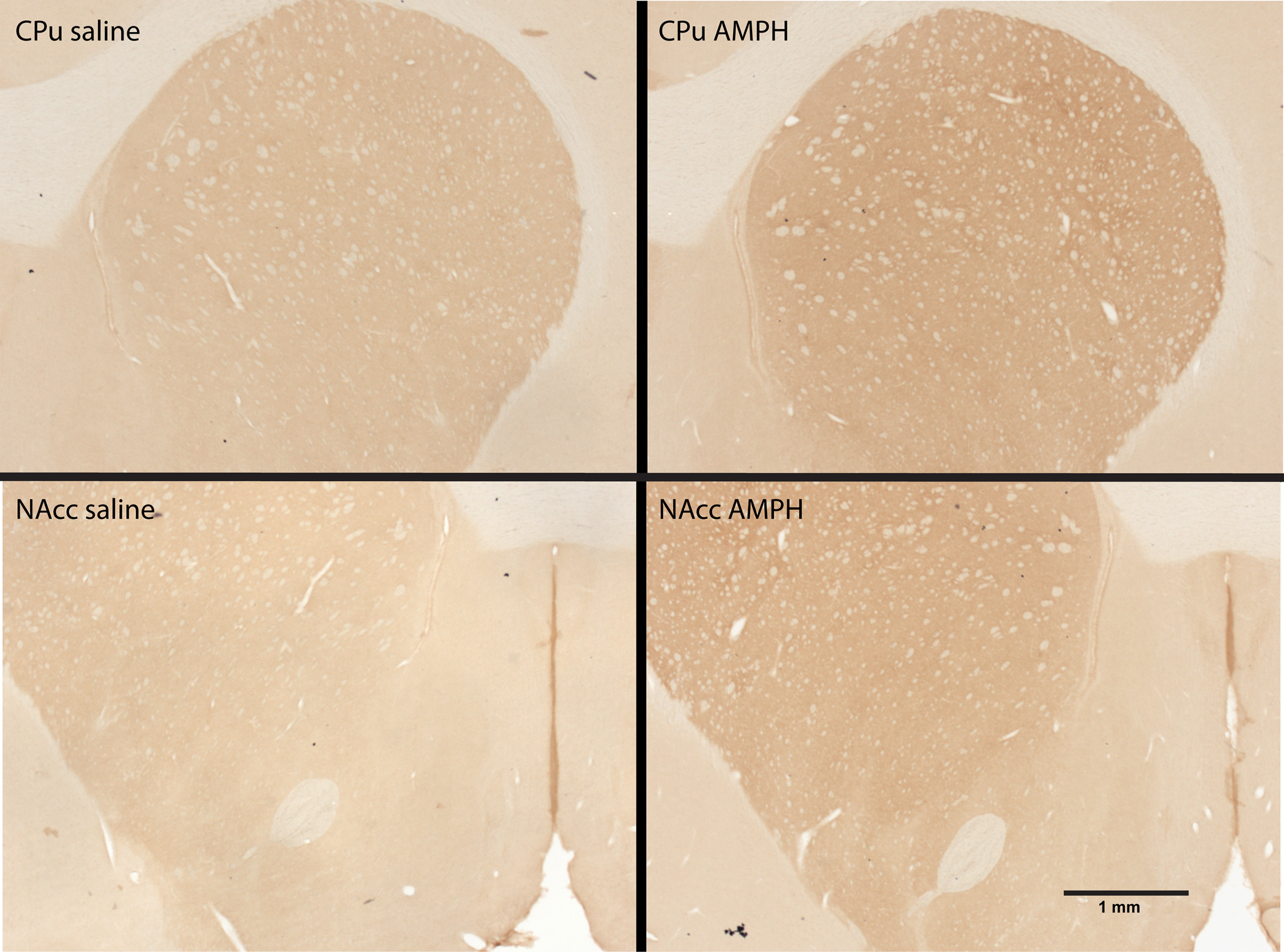

Supplement: S5 Fig — (TIF) [file pone.0172776.s007.tif]

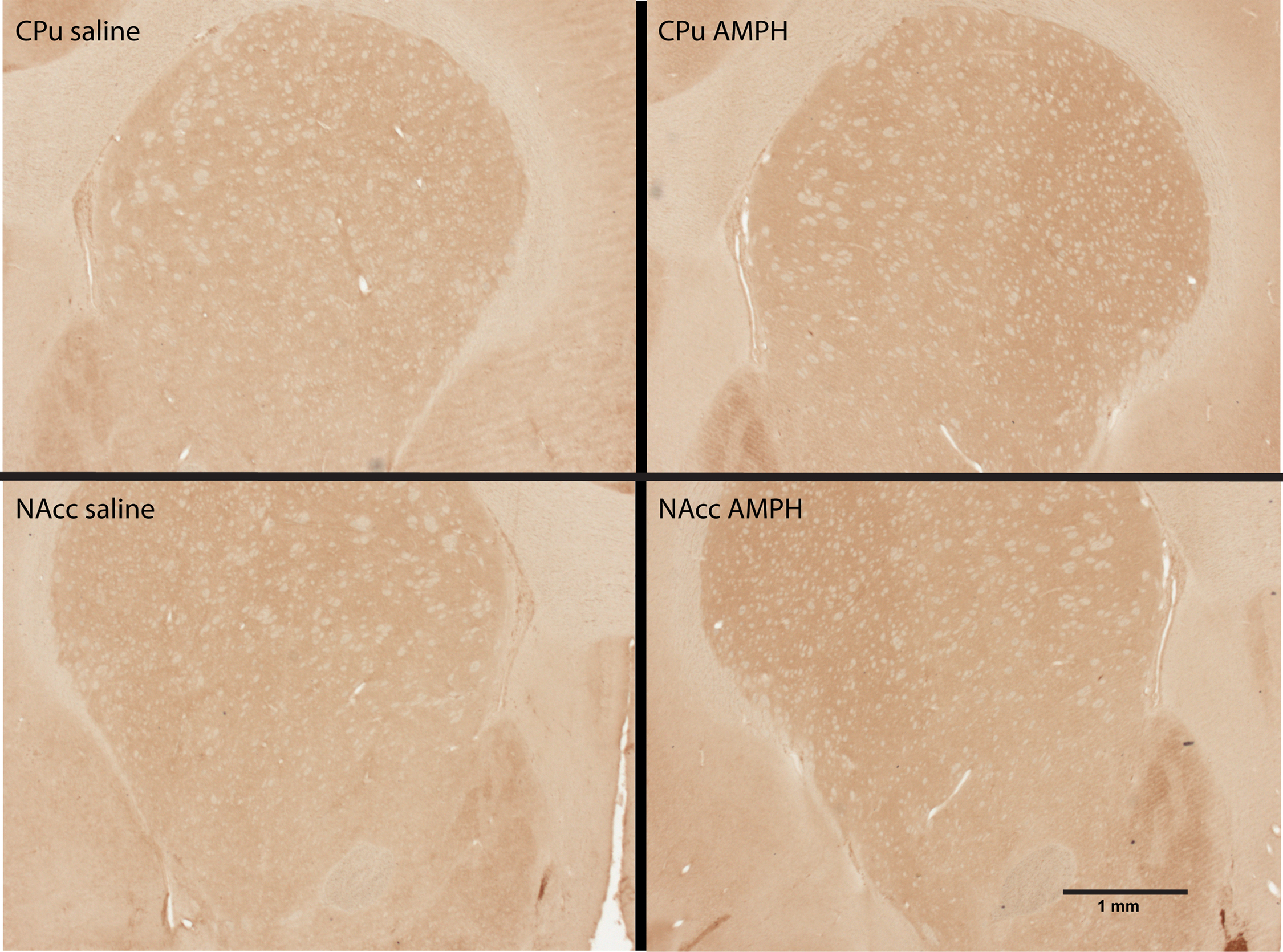

Supplement: S6 Fig — (TIF) [file pone.0172776.s008.tif]

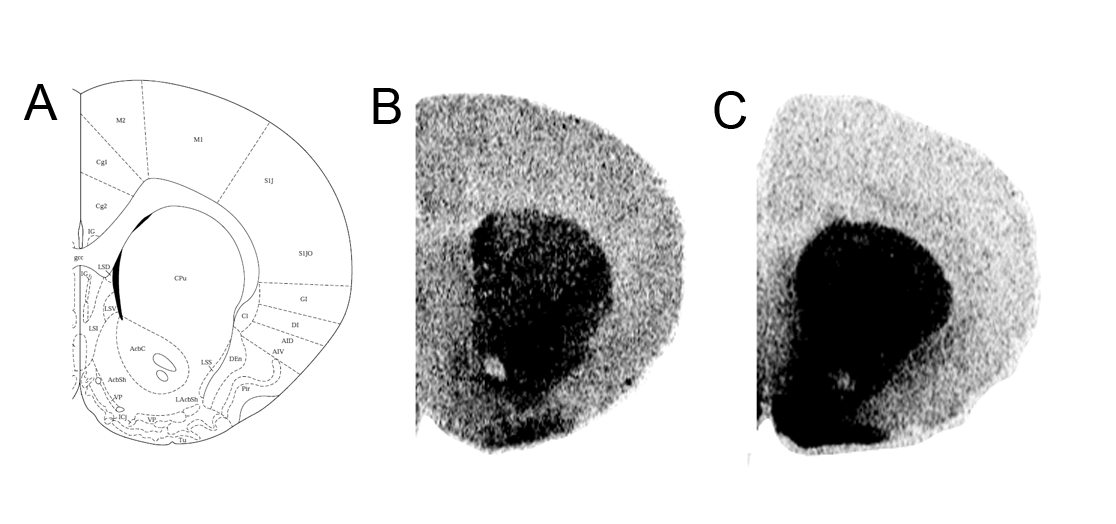

Supplement: S7 Fig — (TIFF) [file pone.0172776.s009.tiff]

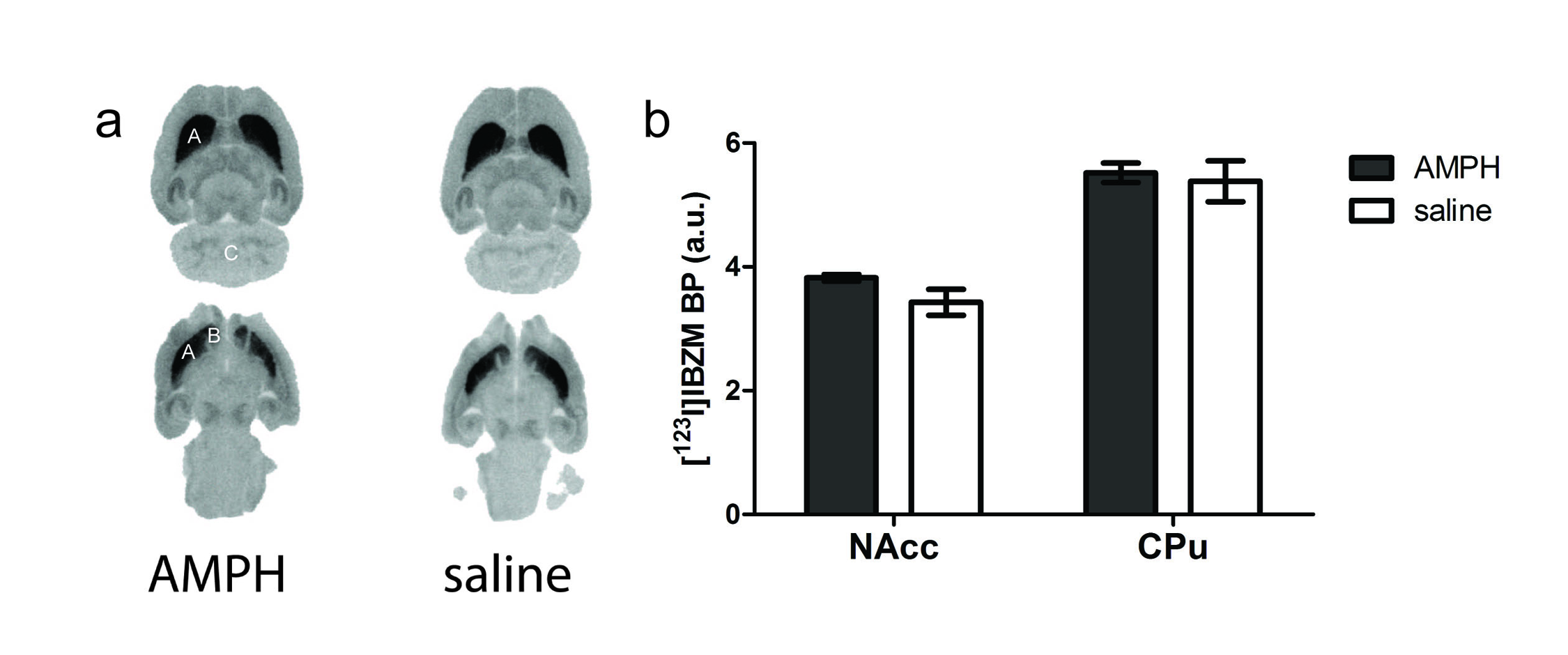

Supplement: S8 Fig — a) Examples of regions of interest for CPu (A), NAcc (B) and cerebellum (C). b) DRD2/3 binding potential in the CPu and NAcc in AMPH and saline pre-treated rats (mean+SEM). (TIF) [file pone.0172776.s010.tif]
